# Seroprevalence of and Risk Factors Associated With SARS-CoV-2 Infection in Health Care Workers During the Early COVID-19 Pandemic in Italy

**DOI:** 10.1001/jamanetworkopen.2021.15699

**Published:** 2021-07-06

**Authors:** Piero Poletti, Marcello Tirani, Danilo Cereda, Giorgio Guzzetta, Filippo Trentini, Valentina Marziano, Claudia Toso, Alessandra Piatti, Raffaella Piccarreta, Alessia Melegaro, Aida Andreassi, Maria Gramegna, Marco Ajelli, Stefano Merler

**Affiliations:** 1 Center for Health Emergencies, Bruno Kessler Foundation, Trento, Italy; 2 Directorate General for Health, Lombardy Region, Milan, Italy; 3 Health Protection Agency of Milan, Italy; 4 Health Protection Agency of Brianza, Monza, Italy; 5 Istituto di Ricovero e Cura a Carattere Scientifico Ca' Granda Ospedale Maggiore Policlinico, Milan, Italy; 6 Dondena Centre for Research on Social Dynamics and Public Policy, Bocconi University, Milan, Italy; 7 Department of Decision Sciences, Bocconi University, Milan, Italy; 8 Department of Social and Political Sciences, Bocconi University, Milan, Italy; 9 Fondazione Istituto di Ricovero e Cura a Carattere Scientifico, Istituto Nazionale dei Tumori, Milan, Italy; 10 Department of Epidemiology and Biostatistics, Indiana University School of Public Health, Bloomington; 11 Laboratory for the Modeling of Biological and Socio-technical Systems, Northeastern University, Boston, Massachusetts

## Abstract

**Importance:**

Identifying health care settings and professionals at increased risk of SARS-CoV-2 infection is crucial to defining appropriate strategies, resource allocation, and protocols to protect health care workers (HCWs) and patients. Moreover, such information is crucial to decrease the risk that HCWs and health care facilities become amplifiers for SARS-CoV-2 transmission in the community.

**Objective:**

To assess the association of different health care professional categories and operational units, including in-hospital wards, outpatient facilities, and territorial care departments, with seroprevalence and odds of SARS-CoV-2 infection.

**Design, Setting, and Participants:**

This cross-sectional study was conducted using IgG serological tests collected from April 1 through May 26, 2020, in the Lombardy region in Italy. Voluntary serological screening was offered to all clinical and nonclinical staff providing any health care services or support to health care services in the region. Data were analyzed from June 2020 through April 2021.

**Exposures:**

Employment in the health care sector.

**Main Outcomes and Measures:**

Seroprevalence of positive IgG antibody tests for SARS-CoV-2 was collected, and odds ratios of experiencing infection were calculated.

**Results:**

A total of 140 782 professionals employed in the health sector were invited to participate in IgG serological screening, among whom 82 961 individuals (59.0% response rate) were tested for SARS-CoV-2 antibodies, with median (interquartile range [IQR]; range) age, 50 (40-56; 19-83) years and 59 839 (72.1%) women. Among these individuals, 10 115 HCWs (12.2%; 95% CI, 12.0%-12.4%) had positive results (median [IQR; range] age, 50 [39-55; 20-80] years; 7298 [72.2%] women). Statistically significantly higher odds of infection were found among health assistants (adjusted odds ratio [aOR], 1.48; 95% CI, 1.33-1.65) and nurses (aOR, 1.28; 95% CI, 1.17-1.41) compared with administrative staff and among workers employed in internal medicine (aOR, 2.24; 95% CI, 1.87-2.68), palliative care (aOR, 1.84; 95% CI, 1.38-2.44), rehabilitation (aOR, 1.59; 95% CI, 1.33-1.91), and emergency departments (aOR, 1.56; 95% CI, 1.29-1.89) compared with those working as telephone operators. Statistically significantly lower odds of infection were found among individuals working in forensic medicine (aOR, 0.40; 95% CI, 0.19-0.88), histology and anatomical pathology (aOR, 0.71; 95% CI, 0.52-0.97), and medical device sterilization (aOR, 0.54; 95% CI, 0.35-0.84) compared with those working as telephone operators. The odds of infection for physicians and laboratory personnel were not statistically significantly different from those found among administrative staff. The odds of infection for workers employed in intensive care units and infectious disease wards were not statistically significantly different from those of telephone operators.

**Conclusions and Relevance:**

These findings suggest that professionals partially accustomed to managing infectious diseases had higher odds of SARS-CoV-2 infection. The findings further suggest that adequate organization of clinical wards and personnel, appropriate personal protective equipment supply, and training of all workers directly and repeatedly exposed to patients with clinical or subclinical COVID-19 should be prioritized to decrease the risk of infection in health care settings.

## Key Points

**Question:**

What are the seroprevalence and the relative odds of SARS-CoV-2 infection among health care workers (HCWs) employed in different professional categories and operational units?

**Findings:**

In this cross-sectional study of 82 961 serological tests of Italian HCWs, 12.2% of participants were positive for IgG antibodies against SARS-CoV-2. Higher odds of infection were found among nurses, health assistants, and workers enrolled in emergency departments or in treatment of patients with subacute disease.

**Meaning:**

These findings suggest that equipment and training of personnel less accustomed to managing infectious disease but directly exposed to patients who may be infected with SARS-CoV-2 should be prioritized to decrease infection risks in health care settings.

## Teaser

This cross-sectional study investigates seroprevalence and odds of SARS-CoV-2 infection among health care workers in different professional categories and operational units in Italy.

## Introduction

Health care workers (HCWs) are essential for the functioning of modern societies. During the first phase of the COVID-19 pandemic, health care professionals were at particularly high risk of SARS-CoV-2 infection, possibly owing to insufficient preparedness for managing a rapid increase in the number of patients seeking primary care and inadequate access to or use of personal protective equipment (PPE).^1,2,3^ The capability of SARS-CoV-2 to be transmitted from individuals who are asymptomatic and presymptomatic^4,5^ likely contributed to increased risk among HCWs, as a consequence of a high number of contacts with undiagnosed infections in clinical settings.

Estimates based on pooled serological studies carried out in different countries suggest that 7% to 8.7% of HCWs were infected by SARS-CoV-2.^6,7^ However, the seroprevalence among people employed in the health sector shows a high variability across countries, ranging from 4% in Denmark to more than 13% in the US.^7,8,9,10,11,12,13^ Heterogeneous infection risks across different categories of hospital wards and health care professions have emerged as well, suggesting that frontline HCWs had greater exposure to SARS-CoV-2 infection compared with nonclinical staff.^6,7,8,11,12,13,14,15,16^ Infection risks in different clinical settings may be associated with how these settings were organized, equipped, and prepared in different geographical areas to face the increase in number of patients during the COVID-19 pandemic. A comprehensive analysis of the relative risk of SARS-CoV-2 infection across different health departments and professional categories is still lacking.

We analyzed the results of an IgG serological screening carried out from April 1 through May 26, 2020, among 82 961 HCWs in the Lombardy region of Italy. With more than 200 000 cumulative identified SARS-CoV-2 infections as of November 3, 2020, Lombardy represents the most and earliest affected Italian region.^17^ This study involved personnel providing direct and indirect patient care, as well as nonclinical staff. This work aimed to identify the health care settings and professions at greatest risk in the initial phase of the pandemic, when health care systems were overburdened by the rapid upsurge in the number of patients with COVID-19. These analyses provide a comprehensive picture of the odds of infection experienced by HCWs employed in hospital wards, outpatient facilities, and territorial care departments in the region in early spring 2020. Our findings may support policy makers and health care and hospital administrators in defining adequate measures to organize clinical settings and ensure appropriate training, resource allocation, and protocols to protect HCWs and patients. These steps may help to decrease risks of SARS-CoV-2 transmission and infection spread from hospitals to wider communities.

## Methods

Data comply with Regulation EU 2016/679 General Data Protection Regulation. Data collection was performed upon informed consent of participants, and the use of these data to assess seroprevalence in the population has been approved by Regione Lombardia (DGR.3114 approved on May 7, 2020, and DGR.3131 approved on May 12, 2020). This cross-sectional study followed the Strengthening the Reporting of Observational Studies in Epidemiology (STROBE) reporting guideline.

### Study Population and Participants

In Italy, health care is provided to all citizens and residents by a mixed public-private system. The public sector is the national health service, which is organized under the Ministry of Health and is administered on a regional basis. Family practice is a division of primary care providing timely, continuing, and comprehensive health care for the individual and family across all ages. In Lombardy, 8 Local Health Units (LHUs) are responsible for territorial care, including primary, ambulatory, domiciliary, and residential care. These units are also responsible for supply services for the prevention of and assistance with chronic diseases and specific pathologies, such as HIV, cancers, and mental disorders.

This analysis focused on the results of a serological screening for IgG antibodies against SARS-CoV-2 targeting all individuals employed in the health care sector in Lombardy. The screening was carried out by regional health authorities as part of outbreak investigations during the COVID-19 pandemic.

The target population consisted of HCWs working in private and public accredited hospitals or research hospitals (Fondazione Istituto di Ricovero e Cura a Carattere Scientifico) or providing any health care service or support to health care services in the region. These individuals included in-hospital HCWs, but also general practitioners and people employed in specialized health care institutions, outpatient facilities, in-home assistance, prehospital emergency medicine, and any territorial service acting as a point of primary, continuing, or palliative care for patients within the regional health care system.

All health care professionals, including physicians, nurses, health care assistants, radiographers, laboratory personnel, paramedics, and administrative and support staff, were invited to participate on a voluntary basis in testing for IgG antibodies against SARS-CoV-2. The term *health assistants* here denotes auxiliary staff who assist patients with personal hygiene, ambulation, dressing, repositioning, feeding, and toileting but who are not certified to change sterile dressings, distribute medications, insert or remove tubing, or conduct tube feedings. Only a fraction of medical students employed in hospitals were invited to participate.

The recruitment of HCWs for serological screening was organized separately by hospital health administrations and, for outpatients’ facilities and general practitioners, by LHUs following a procedure defined by each health directorate. The screening took place from April 1 through May 26, 2020.

As of January 1, 2020, Lombardy’s public hospitals and LHUs employed approximately 88 000 skilled HCWs (including 6016 family doctors and 1162 family pediatricians) and 23 000 nonclinical staff members. From March 8 through May 18, 2020, strict measures were imposed in the region, banning mass gatherings and public events, limiting movement except in case of necessity, and suspending all nonessential productive activities and in-person education in schools and universities. On March 14, 2020, elective surgical treatments, daytime hospital assistance, clinic activities, many screening services (eg, oncological screening), and territorial health care services were suspended in the region because of the COVID-19 pandemic.^18^ These services were gradually reactivated starting on May 7, 2020.^19^ However, none of the HCWs participating in this study were furloughed during the health emergency. On the other hand, new temporary wards were established to manage the sharp increase in the number of patients seeking care. As a consequence, a proportion of employees of the health care sector were reassigned from their duties to support the workload in emergency departments or wards dedicated to low intensity care or treating patients with COVID-19 who had subacute disease or were convalescent.

### Outcome Measures and Data Collection

The test used to detect SARS-CoV-2 IgG antibodies was the Liaison SARS-CoV-2 test (DiaSorin). This test uses magnetic beads coated with S1 and S2 antigens.^20,21^ The antigens used in the test are expressed in human cells to achieve proper folding, oligomer formation, and glycosylation, providing material similar to the native spikes. This strategy ensures that the antigen-antibody complex forms with the required specificity. The S1 and S2 proteins are targets of neutralizing antibodies. The test provides the detection of IgG antibodies against S1 and S2 antigens of SARS-CoV-2 and the detection of neutralizing antibodies with 98.3% specificity and 94.4% sensitivity at 15 days from diagnosis.^20,21^

Data collection, storage, anonymization, and management were carried out by regional health authorities. The same test was applied to all HCWs. Serological samples were analyzed by 37 laboratories hosted in public hospitals of the region that were appropriately equipped to analyze the collected serological samples. Each sample was assigned to a specific laboratory according to proximity to the HCW’s workplace. Test results were binary and communicated to tested participants by their hospital directorate or the LHU administration. Participants were categorized as seropositive if they had developed IgG antibodies. Those who tested positive for IgG antibodies against SARS-CoV-2 were quarantined and tested by a real-time reverse transcription polymerase chain reaction (RT-PCR) assay targeting SARS-CoV-2 genes.^22,23,24^ Individuals with positive RT-PCR results were isolated. Results from RT-PCR were not considered in this analysis.

Data on serological testing were merged with a database providing information on age, sex, professional category, clinical department or operating unit (including hospital wards and medical subspecialties), LHU, and province of employment for all HCWs employed in Lombardy. Serological inconclusive test results were excluded from the analysis.

### Statistical Analysis

The primary outcome of our analysis was the proportion of HCWs in different professional categories and employed in different operational units (ie, in-hospital wards, outpatient facilities, and territorial care departments) with a positive antibody test for SARS-CoV-2. In this analysis, seroprevalence of IgG antibodies among HCWs was evaluated across 8 professional categories (eTable 1 in the Supplement) and 44 health care settings, which reflect the main hospital wards and territorial health care departments in the region (eTable 2 in the Supplement). Specifically, operational units with fewer than 200 employees were aggregated into a single group, excluding subspecialties of internal medicine, for which a threshold of 100 employees was adopted. Wards dedicated to patients with subacute disease, where individuals infected with SARS-CoV-2 were likely hospitalized before receiving laboratory diagnoses, were aggregated with wards dedicated to internal medicine. We also considered 2 separate groups for general practitioners and family pediatricians, given that these groups may have had different levels of exposure to SARS-CoV-2. Departments and operating units consisting entirely of nonclinical employees dedicated to administrative or support services were also aggregated into a unique group. To test robustness to the adopted partition of operational units, we also considered an alternative grouping in which a single operating unit was adopted for all surgical units (ie, general surgical, cardiac surgical, neurosurgical, plastic surgical, urologic, orthopedic, and traumatological units) and a single operating unit was adopted for the following internal medicine subspecialties: cardiology, endocrinology, diabetes, gastroenterology, hepatology, geriatric medicine, hematology, oncology, nephrology, dialysis, dermatology, and rheumatology.

Seroprevalence across different groups was provided as crude percentages, while 95% CIs were computed by exact binomial tests. The association between exposures and IgG results positive for SARS-CoV-2 infection was explored using a generalized linear mixed-effects model with logit link. For the comparisons, administrative staff and telephone operators were considered as the reference groups for the professional categories and operational units, respectively. The rationale behind this choice was that these groups may represents proxies for the general population in terms of risk of SARS-CoV-2 infection. During the first COVID-19 pandemic wave, temporal and cumulative incidence rates were variable across different provinces of Lombardy, and these provinces had different amounts of time to prepare for the upsurge in number of patients. This may have been associated with differences in the management of the COVID-19 crisis among provinces, including whether and how HCWs’ duties were reassigned and temporary COVID-19 wards were set up. Heterogeneities in calendar time of sample collection across specific groups may be associated with the relative odds of positive IgG results across different professional categories. To account for these possible sources of bias, the week of serum collection and a binary variable identifying whether the HCW was employed in a hospital where patients with COVID-19 were more likely to be treated (defined as those facilities equipped with an intensive care unit [ICU] or a ward dedicated to infectious diseases) were considered as possible adjusting factors in the logistic regression. Province-specific random effects were considered to account for heterogeneous exposure to infection in the community and HCWs’ working conditions across different geographical areas. A stepwise selection procedure based on the likelihood ratio test was adopted to compare statistical models considering different covariates. The resulting best model included as covariates the individuals’ sex and age groups, operating units, professional categories, and weeks of serum collection. Adjusted odds ratios (aORs) of SARS-CoV-2 infection were computed conditioning on the covariates. Statistical significance of the parameters of the logistic regression was assessed through a 2-sided Wald test. Statistical significance of differences in aORs was assessed by checking overlaps of aOR CIs with 1. Moran’s index was used to assess spatial autocorrelation in seroprevalence levels across different provinces, using distance from the barycenter of the province or a binary matrix defining the adjacency of different provinces. The statistical analysis was performed using R statistical software version 4.0.2 (R Project for Statistical Computing). Data were analyzed from June 2020 through April 2021.

## Results

Among 140 782 professionals employed in the health sector invited to participate IgG serological screening, 82 961 individuals (59.0% response rate) were tested for SARS-CoV-2 antibodies, with median (interquartile range [IQR]; range) age, 50 [40-56; 19-83] years and 59 839 [72.1%] women. We excluded 1258 participant records owing to inconclusive IgG results, with median (interquartile range [IQR]) age 48 [38-55] years, 927 (73.7%) women, and 821 clinical staff (65.3%) (eFigure in the Supplement). Overall, we analyzed 81 703 HCWs tested from April 1 through June 26, 2020 (median [IQR; range] age, 50 [40-56; 19-83] years; 58 912 [72.1%] women). Of these, there were 29 200 nurses (35.8%), 13 915 physicians (17.0%), 7549 health care assistants (9.2%), 592 radiologists or laboratory personnel (0.7%), and 29 828 administrative or support staff employed in the health care sector (36.5%). There were 1083 general practitioners (1.3%) and 140 family pediatricians (0.2%). A large sample size was obtained in most of the considered provinces (eTable 3 in the Supplement).

**Figure 1.**
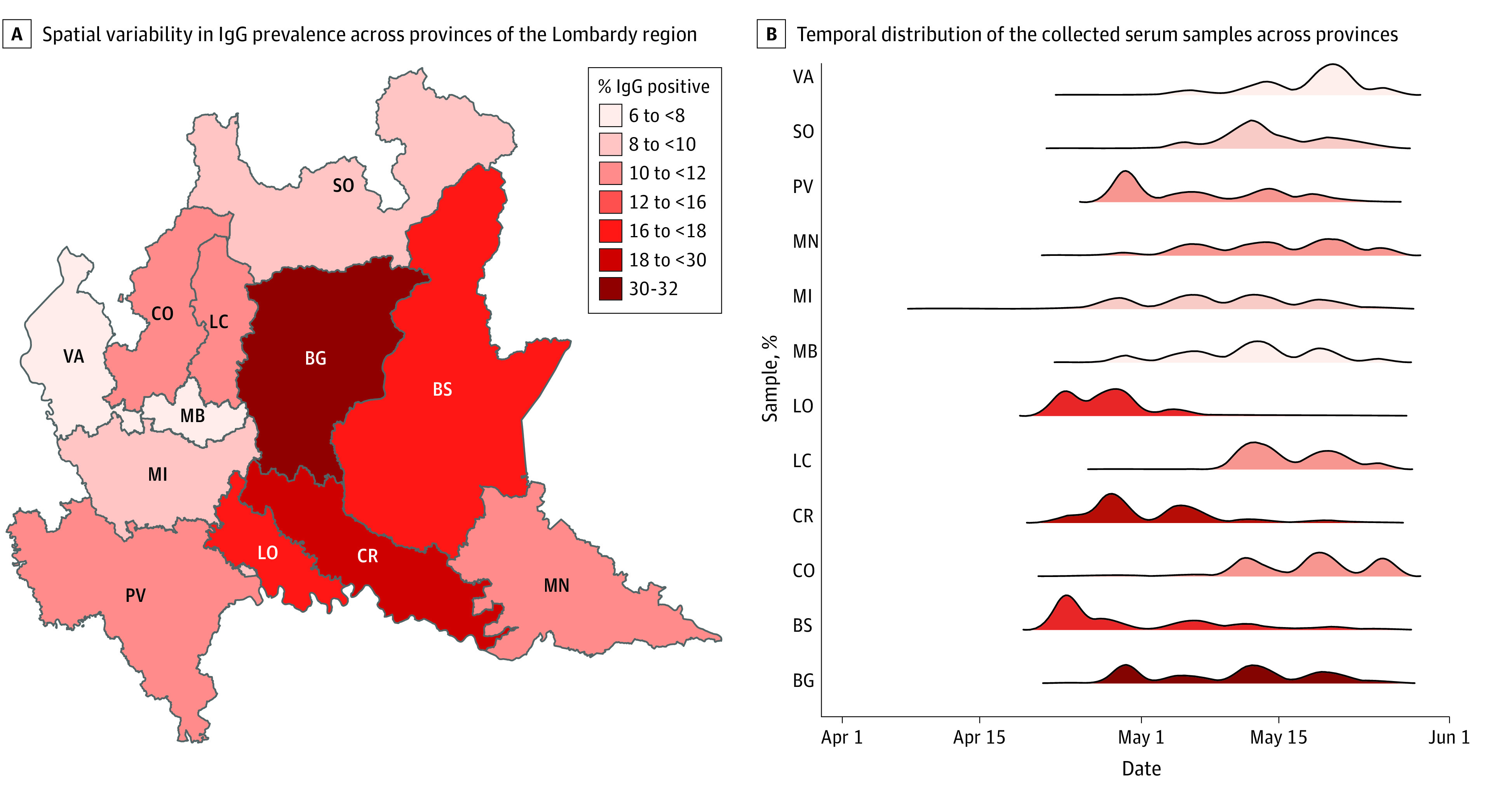
Spatial and Temporal Distribution of Samples A, BG indicates Bergamo; BS, Brescia; CO, Como; colors, the percentage of participants with IgG positive results; CR, Cremona; LC, Lecco; LO, Lodi; MB, Monza-Brianza; MI, Milano; MN, Mantova; PV, Pavia; SO, Sondrio; VA, Varese. B, Colors indicate the percentage of participants with IgG positive results.

**Figure 2.**
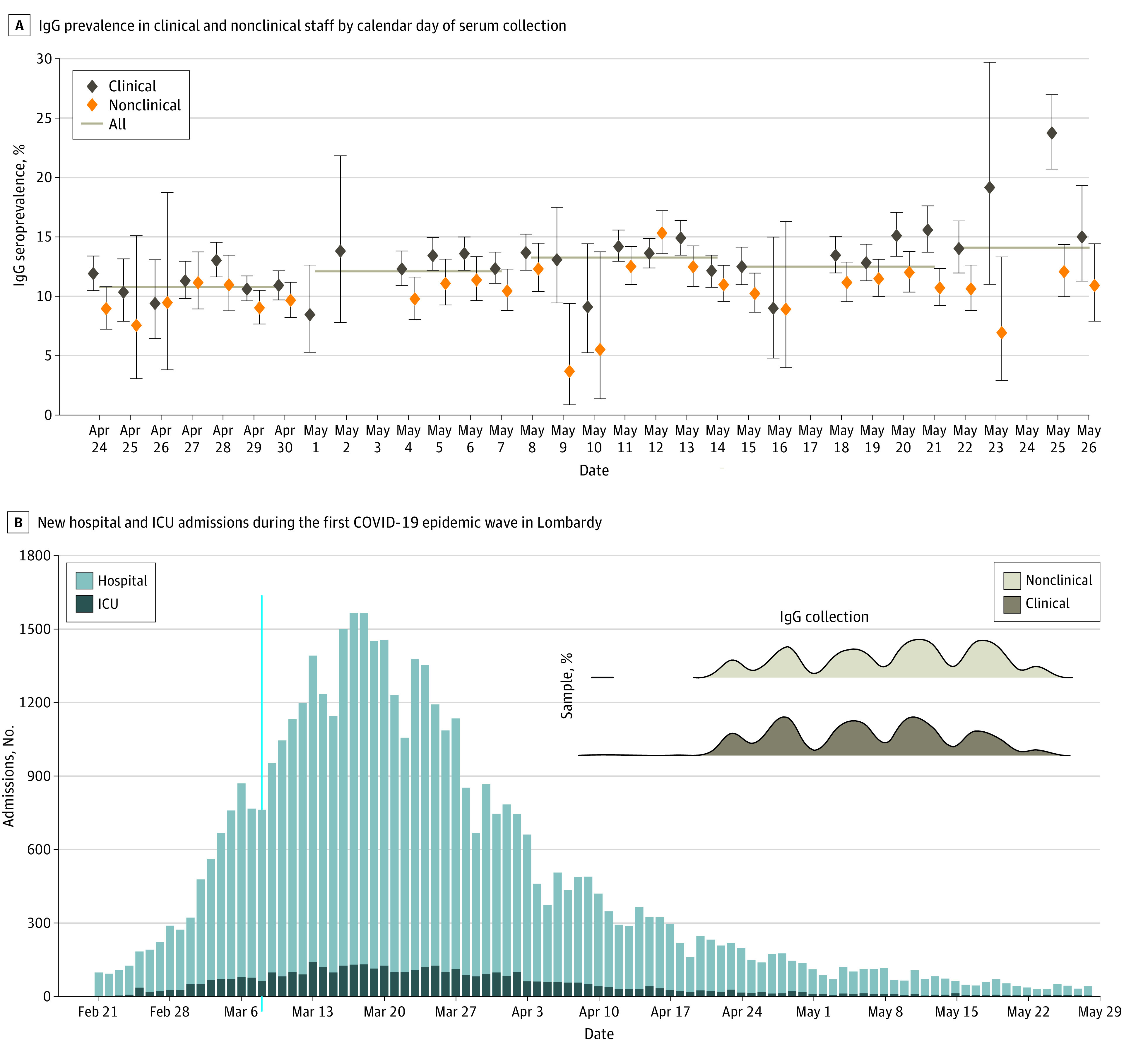
IgG Prevalence and COVID-19 Admissions A, Dots indicate crude point estimates of IgG prevalence in health care workers; vertical lines, 95% CIs computed by exact binomial tests; horizontal lines, weekly means for all study participants (ie, including clinical and nonclinical staff). B, ICU, indicates intensive care unit; shaded area and inset, temporal distribution of collected serum samples in health care workers with clinical and nonclinical disease.

Among 82 961 tested participants, 10 115 HCWs (median [IQR; range] age, 50 [39-55; 20-80] years; 7298 [72.2%] women) developed IgG antibodies against SARS-CoV-2, with an overall seroprevalence of 12.2% (95% CI, 12.0-12.4). The proportion of HCWs with positive results was variable across different provinces, with HCWs working in the most affected provinces having greater exposure to the infection. Seroprevalence among HCWs ranged from 6.7% (95% CI, 6.1%-7.4%) in Monza-Brianza, where there were 31.3 hospitalized patients per 10 000 inhabitants, to 31.3% (95% CI, 30.1%-32.4%) in Bergamo, where there were 78.6 hospitalized patients per 10 000 inhabitants (Pearson correlation: *P* = .003) (Figure 1; eTable 3 in the Supplement). The spatial autocorrelation between seroprevalence levels was not statistically significant considering the adjacency of different provinces or the distance from the barycenter of each province. We tested for an association between the timing of serum collection and a higher IgG seroprevalence among the study participants (Figure 2). However, we did not find an association between different timings of serum collection among study participants and geographical differences in the proportion of HCWs with IgG positive results or the increased proportion of IgG positive results found among clinical staff compared with nonclinical staff (Figure 1 and Figure 2).

**Figure 3.**
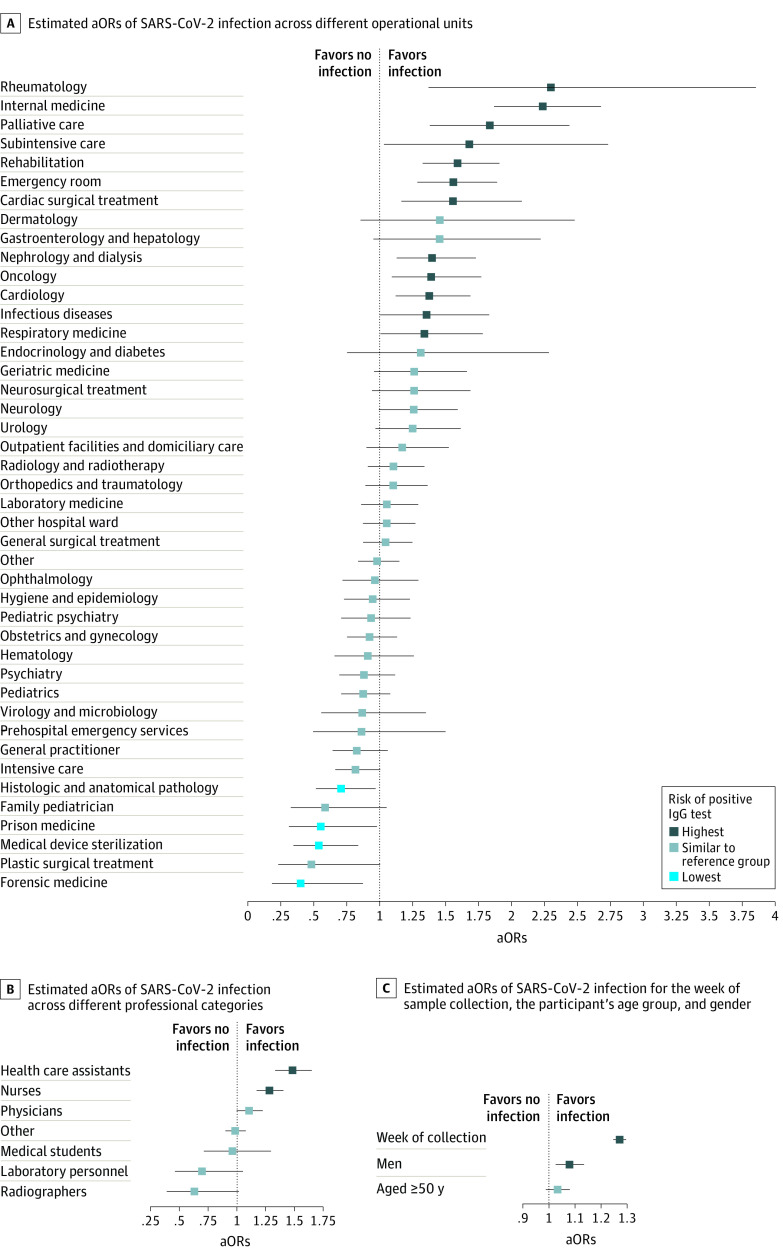
Estimated Adjusted Odds Ratios (aORs) by Operational Unit, Professional Category, and Demographics In panels A, B, and C, dots indicate mean estimates; horizontal lines, 95% CIs; darker colors, higher aORs of positive IgG test results.

Seroprevalence among administrative personnel, representing the reference professional category in our analysis, was 11.6% (95% CI, 10.9-12.3). Compared with the control group, statistically significantly higher odds of infection were found among health assistants (adjusted odds ratio [aOR], 1.48; 95% CI, 1.33-1.65) and nurses (aOR, 1.28; 95% CI, 1.17-1.41), while the odds of infection among physicians were not statistically significantly different (aOR, 1.11; 95% CI, 1.00-1.23) (eTable 1 in the Supplement). Laboratory personnel (aOR, 0.7; 95% CI, 0.46-1.1) and radiologists (aOR, 0.63; 95% CI, 0.39-1.0) had lower odds of developing IgG antibodies, although their relative odds of infection were not statistically significantly different from the control group. Statistically significantly higher odds of infection were found for men (aOR, 1.08; 95% CI, 1.03-1.13) (Figure 3; eTable 4 in the Supplement).

Seroprevalence among telephone operators, who represented the reference operational unit in our analysis, was 11.7% (95% CI, 10.3-13.3). Heterogeneous infection rates were found across different operational units (Figure 3; eTable 2 in the Supplement). The highest aORs of infection were found among people employed in internal medicine (aOR, 2.24; 95% CI, 1.87-2.68) and rheumatology (aOR, 2.30; 95% CI, 1.37-3.86), followed by those employed in palliative care (aOR, 1.84; 95% CI, 1.38-2.44), rehabilitation (aOR, 1.59; 95% CI, 1.33-1.91), emergency departments (aOR, 1.56; 95% CI, 1.29-1.89), cardiac surgical units (aOR, 1.56; 95% CI, 1.17-2.08), nephrology or dialysis (aOR, 1.40 95% CI, 1.13-1.73), and cardiology (aOR, 1.38; 95% CI, 1.12-1.69). Although the adjusted odds of infection were not statistically significantly different from the control group, high seroprevalence was also found in geriatric medicine (17.2%; 95% CI, 14.2%-20.5%), urology (16.0%; 95% CI, 13.5%-18.7%), and outpatient facilities or domiciliary care (18.2%; 95% CI, 15.2%-21.5%).

The lowest aORs of infection were found among personnel working in forensic medicine (aOR, 0.40; 95% CI, 0.19-0.88), histology and anatomical pathology (aOR, 0.71; 95% CI, 0.52-0.97), and medical device sterilization (aOR, 0.54; 95% CI, 0.35-0.84). Although the adjusted odds of infection were not statistically significantly different from the control group, low seroprevalence levels were also found among HCWs employed in ICUs (8.2%; 95% CI, 7.2%-9.2%), pediatrics (8.8%; 95% CI, 7.7%-9.9%), plastic surgical units (4.8%; 95% CI, 2.1%-9.2%), hematology (7.5%; 95% CI, 5.7%-9.7%), and virology or microbiology (7.8%; 95% CI, 5.1%-11.4%). Although the odds of infection among HCWs employed in infectious diseases were statistically significantly higher than those found among telephone operators (aOR, 1.36; 95% CI, 1.05-1.83), similar seroprevalence levels were found in professionals working in these 2 units (11.9%; 95% CI, 9.4%-14.8% vs 11.7%; 95% CI, 10.3%-13.3%).

## Discussion

This cross-sectional study of serological screening conducted in Lombardy during the COVID-19 pandemic found that at the end of May 2020 approximately 12% of people employed in the health sector tested positive for IgG antibodies against SARS-CoV-2, with this value ranging from 6% to 31% depending on the province considered. The spatial variability we found in HCWs’ odds of infection may be associated with the different infection rates across different geographical areas and the spatiotemporal spread of SARS-CoV-2 during the initial pandemic phase.^25^ These differences are associated with the underlying probability among HCWs of getting infected outside their workplaces (eg, at home) and with the number of patients who were infectious who were assisted at work (eTable 3 in the Supplement). We found higher odds of infection among HCWs employed in areas with greater infection rates. These areas were also the first to be hit by the pandemic, and their health care systems thus had less time to prepare (eg, in terms of PPE supply and hygiene protocols) for the ensuing rapid increase in number of hospitalized patients. The association of SARS-CoV-2 seropositivity among health care personnel with COVID-19 incidence in the community was recently highlighted by a serological study^26^ comparing seroprevalence levels found in 4 large health care systems located in 3 states in the US.

In our analysis, we found that HCWs employed in hospital emergency departments or enrolled in treatment of patients with subacute and subclinical disease had higher aORs of SARS-CoV-2 infection than health professionals working in ICUs or wards dedicated to infectious diseases or managing patients’ specimens or employed in the sterilization of medical devices. These findings suggest that health care professionals who were partially accustomed to managing infectious disease cases but who may have been directly exposed to patients with COVID-19 may have been at high risk of SARS-CoV-2 infection.

In addition to the higher odds of infection found among men, we found that health assistants and nurses had higher aORs of infection than physicians. This result suggests that repeated and prolonged contacts with patients who were infected represented a significant risk factor associated with SARS-CoV-2 positivity among HCWs. A higher seroprevalence among health care assistants compared with physicians was also found in a SARS-CoV-2 serosurvey involving 8285 HCWs in the Veneto region,^27^ where detectable antibodies were found in 4.6% of participants.

Epidemiological evidence from the analysis of contact tracing data suggests that transmissibility is not associated with the severity of disease.^4^ However, to our knowledge, no specific study is available assessing whether there is an association between disease severity and risk of nosocomial transmission. In principle, it is possible that HCWs assisting patients requiring additional care may have a higher risk of infection. According to our results, people employed in ICUs had odds of infection similar to those of telephone operators. This finding suggests that appropriate equipment and adherence to hygiene protocols may represent valuable strategies for protecting HCWs and may prevent transmission in health care settings. The relative risk of infection experienced in ICUs with respect to other hospital wards should be cautiously considered, given that it is associated with the number of admissions and the adopted protocols (including the use of PPE) at different levels of intensity of care. A relatively lower risk of infection in ICUs was also found in the New York City area and in countries where the serological prevalence among HCWs was 7% to 24%.^6,11,12,28^ The lower risk of infection of pediatricians and the higher risk identified for nurses, health assistants and HCWs employed in emergency department has been already highlighted in a number of countries.^6,7,8,11,12^ In particular, the lower aORs of infection we found among pediatricians compared with HCWs employed in geriatric medicine is clearly associated with the age distribution of patients with COVID-19 seeking care, which was skewed toward older ages (eTable 5 in the Supplement). On the other hand, the high aORs of infection in the emergency department may be associated with the frequent overcrowding observed in these settings^29^ combined with high exposure to patients with severe disease and silent transmission from individuals without symptoms (including those who are presymptomatic or asymptomatic).^4,5,30,31^

A peculiarity that emerged in this study was the higher infection aORs characterizing people working in internal medicine and related subspecialties (eg, rheumatology), who may have supported health care services in COVID-19 wards during the period of higher pressure on the health care system. This statistical outcome was confirmed when aggregating internal medicine subspecialties into a single operating unit (eTables 6-8 in the Supplement) but contrasts with the low risk of infection identified for these wards in Denmark.^8^ However, in Denmark, 4% of HCWs were positive for SARS-CoV-2 and the development of the pandemic was less dramatic, with more time for preparation and ensuring sufficient access to PPE compared with Italy.^8^ Therefore, our findings may be associated with the way hospital wards and personnel were reorganized in Lombardy to face the rapid increase in numbers of patients with COVID-19. This may be the case for the high aORs of infection we found in departments dedicated to rehabilitation, where patients with COVID-19 who were convalescent may have been hosted when the health system was under significant strain. Similar arguments may apply to personnel working in cardiac surgical units, who may have been employed in wards dedicated to low intensity of care after the suspension of surgical activities on March 14, 2020. These results suggest that when the health care system in Lombardy was operating at its maximum capacity to better face the pandemic threat, a remarkable proportion of health care personnel less experienced in treating infectious diseases were exposed to a high number of patients with SARS-CoV-2. The higher aORs of infection we found among these professionals may therefore have been associated with clinical settings and personnel insufficiently prepared or equipped to treat a huge number of patients.

On January 22, 2020, the Italian Ministry of Health and the Lombardy region provided instructions to LHUs and hospitals for ensuring the PPE supply and distribution along with appropriate training of all personnel at risk of infection. However, shortages in PPE were reported during the early phase of the pandemic, especially in non-COVID wards,^2,6,7^ resulting in usage of the equipment for prolonged periods compared with those recommended by defined protocols and differential distributions to personnel less exposed to confirmed SARS-CoV-2 infections.^1,3^ This may partially explain the high aORs of infection found among employees of palliative care units. Additionally, a low seroprevalence of IgG antibodies was found in HCWs working in hematology compared with other subspecialties of internal medicine. This may be associated with the attention devoted to hygiene protocols to protect patients at greatest risk.^14^

Although in some cases no workplace factors were found to be associated with SARS-CoV-2 seropositivity of health care personnel,^26^ a recent meta-analysis found that being male, being a health care assistant, working in a COVID-19 unit, directly assisting patients, and shortages in PPE were common factors associated with increased risk of infection among HCWs.^7^

### Limitations

This study has several limitations. The imperfect sensitivity (94.4% sensitivity at 15 days from diagnosis) and specificity (98.3%) of the assays used to identify SARS-CoV-2 infection in this study should be taken into account when interpreting the results. The impact of false negatives owing to delays in seroconversion should be limited,^20^ but temporal waning of IgG antibodies among asymptomatic infections cannot be excluded.^13,32^ Data concerning participants’ antibody levels and symptoms, underlying health conditions, and contacts with individuals infected with SARS-CoV-2 (within and outside their workplace) would have been beneficial to provide a more comprehensive picture of SARS-CoV-2 infection among HCWs. In particular, we were not able to pinpoint whether the source of infection among study participants was their workplace (eg, contacts with patients or colleagues), their household, or other contacts. Although a significantly higher seroprevalence was found among HCWs employed in provinces where an increased number of COVID-19 hospital admissions were recorded, no information was available concerning which hospital wards and health departments were dedicated to treating patients with COVID-19. This limitation prevented us from evaluating the role played by the number of admitted patients across different hospital wards and by potential outbreaks occurring in participant workplaces. Additionally, the lack of data on PPE adoption among HCWs prevented us from evaluating the association of precautionary behavior (or PPE supply) over time and across different departments with the odds of infection.

## Conclusions

In this cross-sectional study of HCWs in Italy, experiencing direct and prolonged contacts with individuals who were infected, rather than exposure to the most severe infections or specimens from individuals who were infected, emerged as risk factors associated with SARS-CoV-2 infection among health care professionals. The highest aORs of infection were found among health assistants, nurses, and people working in emergency departments, palliative care units, and hospital wards dedicated to patients who had subacute or subclinical disease or were convalescent. Risk factors associated with SARS-CoV-2 infection among HCWs may include inadequate PPE supply and inappropriate protocols while caring for patients, which may be more likely to occur among personnel less accustomed to treating infectious diseases or in overcrowded hospitals with high numbers of patients with COVID-19. These results suggest that adequate measures are needed to organize clinical settings; ensure training, support, and provision of PPE for all health care personnel treating patients with clinical COVID-19; and prevent potential transmission from nonclinical infections. Adherence to hygiene protocols and the maintenance of universal masking among HCWs remain necessary ingredients in a multipronged strategy for infection reduction in health care settings.^9^ This analysis highlighted those health care professionals and clinical settings that had greater risk of SARS-CoV-2 during the first COVID-19 pandemic wave, when the Italian heath system was overburdened. It is thus important to stress that the estimated risk factors may not reflect the situation in other phases of the pandemic or in other countries.

## Notes

Supplement. **eFigure.** Flowchart of Inclusions and Exclusions **eTable 1.** Seroprevalence and Odds Ratios of Infection by Professional Category **eTable 2.** Seroprevalence and Odds Ratios of Infection by Operational Unit **eTable 3.** Seroprevalence of Infection and Overall Hospitalization Incidence by Province, February to September 2020 **eTable 4.** Seroprevalence and Odds Ratios of Infection by Age Group and Sex **eTable 5.** Hospital Admission by Age Group, February to September 2020 **eTable 6.** Seroprevalence and Odds Ratios by Professional Category Considering 1 Overall Surgical Unit and 1 Overall Internal Medicine Subspeciality Unit **eTable 7.** Seroprevalence and Odds Ratios by Age Group and Sex Considering 1 Overall Surgical Unit and 1 Overall Internal Medicine Subspeciality Unit **eTable 8.** Seroprevalence and Odds Ratios by Operational Unit Considering 1 Overall Surgical Unit and 1 Overall Internal Medicine Subspeciality Unit

## References

[1] Nguyen LH, Drew DA, Graham MS, ; Coronavirus Pandemic Epidemiology Consortium. Risk of COVID-19 among front-line health-care workers and the general community: a prospective cohort study. Lancet Public Health. 2020;5(9):e475-e483. Published online July 31, 2020. doi:10.1016/S2468-2667(20)30164-X 32745512 PMC7491202

[2] Ranney ML, Griffeth V, Jha AK. Critical supply shortages —the need for ventilators and personal protective equipment during the COVID-19 pandemic. N Engl J Med. 2020;382(18):e41. Published online March 25, 2020. doi:10.1056/NEJMp2006141 32212516

[3] Leung NHL, Chu DKW, Shiu EYC, . Respiratory virus shedding in exhaled breath and efficacy of face masks. Nat Med. 2020;26(5):676-680. doi:10.1038/s41591-020-0843-2 32371934 PMC8238571

[4] Hu S, Wang W, Wang Y, Litvinova M, Luo K, Ren L, Infectivity, susceptibility, and risk factors associated with SARS-CoV-2 transmission under intensive contact tracing in Hunan, China. medRxiv. Preprint posted online November 3, 2020. doi:10.1101/2020.07.23.20160317 PMC7943579 33750783

[5] Szablewski CM, Chang KT, Brown MM, SARS-CoV-2 transmission and infection among attendees of an overnight camp—Georgia, June 2020. MMWR Morb Mortal Wkly Rep. 2020;69(31):362-365. doi:10.15585/mmwr.mm6931e1 PMC7454898 32759921

[6] Gómez-Ochoa SA, Franco OH, Rojas LZ, . COVID-19 in health-care workers: a living systematic review and meta-analysis of prevalence, risk factors, clinical characteristics, and outcomes. Am J Epidemiol. 2021;190(1):161-175. doi:10.1093/aje/kwaa191 32870978 PMC7499478

[7] Galanis P, Vraka I, Fragkou D, Bilali A, Kaitelidou D. Seroprevalence of SARS-CoV-2 antibodies and associated factors in healthcare workers: a systematic review and meta-analysis. J Hosp Infect. 2021;108:120-134. doi:10.1016/j.jhin.2020.11.008 33212126 PMC7668234

[8] Iversen K, Bundgaard H, Hasselbalch RB, . Risk of COVID-19 in health-care workers in Denmark: an observational cohort study. Lancet Infect Dis. 2020;20(12):1401-1408. doi:10.1016/S1473-3099(20)30589-2 32758438 PMC7398038

[9] Wang X, Ferro EG, Zhou G, Hashimoto D, Bhatt DL. Association between universal masking in a health care system and SARS-CoV-2 positivity among health care workers. JAMA. 2020;324(7):703-704. doi:10.1001/jama.2020.12897 32663246 PMC7362190

[10] Houlihan C, Vora N, Byrne T, Lewer D, Heaney J, Moore DA, SARS-CoV-2 virus and antibodies in front-line health care workers in an acute hospital in London: preliminary results from a longitudinal study. medRxiv. Preprint posted online June 9, 2020. doi:10.1101/2020.06.08.20120584

[11] Moscola J, Sembajwe G, Jarrett M, ; Northwell Health COVID-19 Research Consortium. Prevalence of SARS-CoV-2 antibodies in health care personnel in the New York City area. JAMA. 2020;324(9):893-895. doi:10.1001/jama.2020.14765 32780804 PMC7411936

[12] Jeremias A, Nguyen J, Levine J, . Prevalence of SARS-CoV-2 infection among health care workers in a tertiary community hospital. JAMA Intern Med. 2020;180(12):1707-1709. doi:10.1001/jamainternmed.2020.4214 32780100 PMC7420823

[13] Calcagno A, Ghisetti V, Emanuele T, . Risk for SARS-CoV-2 infection in healthcare workers, Turin, Italy. Emerg Infect Dis. 2021;27(1):303-305. doi:10.3201/eid2701.203027 33021927 PMC7774556

[14] World Health Organization. Mask use in the context of COVID-19: interim guidance. Accessed May 26, 2021. https://www.who.int/publications/i/item/advice-on-the-use-of-masks-in-the-community-during-home-care-and-in-healthcare-settings-in-the-context-of-the-novel-coronavirus-(2019-ncov)-outbreak

[15] World Health Organization. Transmission of SARS-CoV-2: implications for infection prevention precautions: scientific brief. Accessed May 26, 2021. https://www.who.int/news-room/commentaries/detail/transmission-of-sars-cov-2-implications-for-infection-prevention-precautions

[16] Ferioli M, Cisternino C, Leo V, Pisani L, Palange P, Nava S. Protecting healthcare workers from SARS-CoV-2 infection: practical indications. Eur Respir Rev. 2020;29(155):200068. doi:10.1183/16000617.0068-2020 32248146 PMC7134482

[17] Ministero della Salute. COVID-19 Situazione in Italia. Accessed May 26, 2021. https://www.salute.gov.it/portale/nuovocoronavirus/dettaglioContenutiNuovoCoronavirus.jsp?area=nuovoCoronavirus&id=5351&lingua=italiano&menu=vuoto

[18] Regione Lombardia. Decreto N. 3351 del 14/03/2020: disposizioni integrative in attuazione della DGR N. XI/2906 DELL’8/03/2020 per l’organizzazione della rete ospedaliera in ordine all’emergenza epidemiologica da COVID-19. Accessed May 26, 2021. https://www.osservatorionazionalescreening.it

[19] Regione Lombardia. Delibera n. 3115 del 7 maggio 2020—indirizzi per l'organizzazione delle attività sanitarie. Accessed May 26, 2021. https://www.regione.lombardia.it/wps/wcm/connect/0409d41a-e474-49cd-af65-1c2250ba07ab/DGR+n_+3115_07_05_2020.pdf?MOD=AJPERES&CACHEID=ROOTWORKSPACE-0409d41a-e474-49cd-af65-1c2250ba07ab-n8eAvAA

[20] Bonelli F, Sarasini A, Zierold C, . Clinical and analytical performance of an automated serological test that identifies S1/S2-neutralizing IgG In COVID-19 patients semiquantitatively. 2020. J Clin Microbiol. 2020;58(9):01224-20. doi:10.1128/JCM.01224-20 32580948 PMC7448652

[21] GeurtsvanKessel CH, Okba NMA, Igloi Z, Towards the next phase: evaluation of serological assays for diagnostics and exposure assessment. medRxiv. Preprint posted online May 5, 2020. doi:10.1101/2020.04.23.20077156

[22] Cereda D, Tirani M, Rovida F, The early phase of the COVID-19 outbreak in Lombardy, Italy. arXiv. Preprint posted March 20, 2020. Accessed May 26, 2021. https://arxiv.org/abs/2003.09320

[23] Corman VM, Landt O, Kaiser M, . Detection of 2019 novel coronavirus (2019-nCoV) by real-time RT-PCR. Euro Surveill. 2020;25(3):2000045. doi:10.2807/1560-7917.ES.2020.25.3.2000045 31992387 PMC6988269

[24] Cohen AN, Kessel B. False positives in reverse transcription PCR testing for SARS-CoV-2. medRxiv. Preprint posted online May 1, 2020. Accessed May 26, 2021. https://www.medrxiv.org/content/10.1101/2020.04.26.20080911v1

[25] Istituto Superiore di Sanità. COVID-19 integrated surveillance: key national data. Accessed November 5, 2020. https://www.epicentro.iss.it/en/coronavirus/sars-cov-2-integrated-surveillance-data

[26] Jacob JT, Baker JM, Fridkin SK, . Risk factors associated with SARS-CoV-2 seropositivity among US health care personnel. JAMA Netw Open. 2021;4(3):e211283. doi:10.1001/jamanetworkopen.2021.1283 33688967 PMC7948059

[27] Plebani M, Padoan A, Fedeli U, . SARS-CoV-2 serosurvey in health care workers of the Veneto region. Clin Chem Lab Med. 2020;58(12):2107-2111. doi:10.1515/cclm-2020-1236 32845861

[28] Shields A, Faustini SE, Perez-Toledo M, . SARS-CoV-2 seroprevalence and asymptomatic viral carriage in healthcare workers: a cross-sectional study. Thorax. 2020;75(12):1089-1094. doi:10.1136/thoraxjnl-2020-215414 32917840 PMC7462045

[29] O’Dowd A. Emergency departments must not return to pre-covid days of overcrowding and lack of safety, says college. BMJ. 2020;369:m1848. doi:10.1136/bmj.m1848 32376614

[30] Poletti P, Tirani M, Cereda D, Probability of symptoms and critical disease after SARS-CoV-2 infection. arXiv. Preprint posted online June 15, 2020. Accessed May 26, 2021. https://arxiv.org/abs/2006.08471

[31] Stein-Zamir C, Abramson N, Shoob H, . A large COVID-19 outbreak in a high school 10 days after schools’ reopening, Israel, May 2020. Euro Surveill. 2020;25(29):2001352. doi:10.2807/1560-7917.ES.2020.25.29.2001352 32720636 PMC7384285

[32] Long QX, Tang XJ, Shi QL, . Clinical and immunological assessment of asymptomatic SARS-CoV-2 infections. Nat Med. 2020;26(8):1200-1204. doi:10.1038/s41591-020-0965-6 32555424

