# Supplementary material for: Seroprevalence of and Risk Factors Associated With SARS-CoV-2 Infection in Health Care Workers During the Early COVID-19 Pandemic in Italy
Source: JAMA Netw Open. 2021 Jul 6;4(7):e2115699. doi: 10.1001/jamanetworkopen.2021.15699 (PMC8261609; doi:10.1001/jamanetworkopen.2021.15699)
Supplement: Supplement. — eFigure. Flowchart of Inclusions and Exclusions eTable 1. Seroprevalence and Odds Ratios of Infection by Professional Category eTable 2. Seroprevalence and Odds Ratios of Infection by Operational Unit eTable 3. Seroprevalence of Infection and Overall Hospitalization Incidence by Province, February to September 2020 eTable 4. Seroprevalence and Odds Ratios of Infection by Age Group and Sex eTable 5. Hospital Admission by Age Group, February to September 2020 eTable 6. Seroprevalence and Odds Ratios by Professional Category Considering 1 Overall Surgical Unit and 1 Overall Internal Medicine Subspeciality Unit eTable 7. Seroprevalence and Odds Ratios by Age Group and Sex Considering 1 Overall Surgical Unit and 1 Overall Internal Medicine Subspeciality Unit eTable 8. Seroprevalence and Odds Ratios by Operational Unit Considering 1 Overall Surgical Unit and 1 Overall Internal Medicine Subspeciality Unit [file jamanetwopen-e2115699-s001.pdf]

Supplemental Online Content

Poletti P, Tirani M, Cereda D. Seroprevalence of and risk factors associated with SARS-CoV-2 infection in health care workers during the early COVID-19 pandemic in Italy. *JAMA Netw Open*. 2021;4(7):e2115699.
doi:10.1001/jamanetworkopen.2021.15699

eFigure. Flowchart of Inclusions and Exclusions
eTable 1. Seroprevalence and Odds Ratios of Infection by Professional Category
eTable 2. Seroprevalence and Odds Ratios of Infection by Operational Unit
eTable 3. Seroprevalence of Infection and Overall Hospitalization Incidence by Province, February to September 2020
eTable 4. Seroprevalence and Odds Ratios of Infection by Age Group and Sex
eTable 5. Hospital Admission by Age Group, February to September 2020
eTable 6. Seroprevalence and Odds Ratios by Professional Category Considering 1 Overall Surgical Unit and 1 Overall Internal Medicine Subspeciality Unit
eTable 7. Seroprevalence and Odds Ratios by Age Group and Sex Considering 1 Overall Surgical Unit and 1 Overall Internal Medicine Subspeciality Unit
eTable 8. Seroprevalence and Odds Ratios by Operational Unit Considering 1 Overall Surgical Unit and 1 Overall Internal Medicine Subspeciality Unit

This supplemental material has been provided by the authors to give readers additional information about their work.

© 2021 Poletti P et al. *JAMA Network Open.*

eFigure. Flowchart of Inclusions and Exclusions

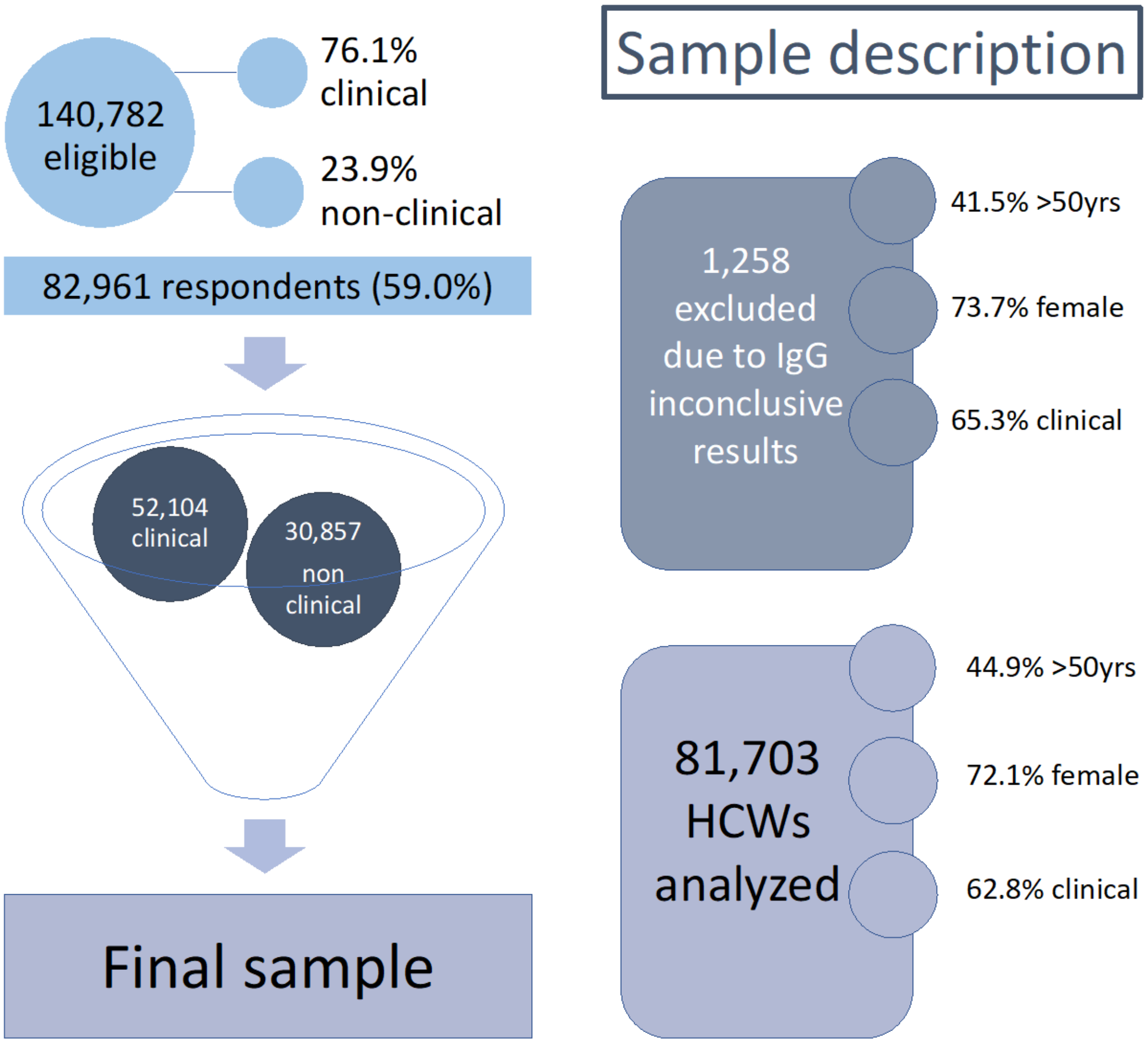

© 2021 Poletti P et al. *JAMA Network Open.*

eTable 1. Seroprevalence and Odds Ratios of Infection by Professional Category

Professional category	Tested for IgG antibodies	IgG positive	Seroprevalence	aOR of infection¶	cOR of infection†
Administrative	8135	940	11.6% (95%CI: 10.9-12.3)	reference	reference
Healthcare assistants	7549	1184	15.7% (95%CI: 14.9-16.5)	1.48 (95%CI: 1.33-1.65)	1.42 (95%CI: 1.3-1.56)
Nurses	29200	3905	13.4% (95%CI: 13.0-13.8)	1.28 (95%CI: 1.17-1.41)	1.18 (95%CI: 1.1-1.28)
Physicians	13915	1575	11.3% (95%CI: 10.8-11.9)	1.11 (95%CI: 1.00-1.23)	0.98 (95%CI: 0.90-1.06)
Medical students	619	55	8.89% (95%CI: 6.76-11.4)	0.96 (95%CI: 0.713-1.3)	0.75 (95%CI: 0.56-0.98)
Laboratory personnel	346	28	8.09% (95%CI: 5.44-11.5)	0.70 (95%CI: 0.46-1.05)	0.67 (95%CI: 0.45-0.98)
Radiographers	246	21	8.54% (95%CI: 5.36-12.8)	0.63 (95%CI: 0.39-1.02)	0.71 (95%CI: 0.44-1.10)
Other	21693	2407	11.1% (95%CI: 10.7-11.5)	0.99 (95%CI: 0.90-1.08)	0.96 (95%CI: 0.88-1.04)

¶ estimates adjusted for model covariates

† crude estimates

© 2021 Poletti P et al. *JAMA Network Open.*

eTable 2. Seroprevalence and Odds Ratios of Infection by Operational Unit

Operational unit	Tested for IgG antibodies	IgG positive	Seroprevalence	aOR of infection¶	cOR of infection†
Telephone operators	1819	213	11.7% (95%CI: 10.3-13.3)	reference	reference
Rheumatology	108	20	18.5% (95%CI: 11.7-27.1)	2.30 (95%CI: 1.37-3.86)	1.71 (95%CI: 1.01-2.79)
Internal medicine	3598	838	23.3% (95%CI: 21.9-24.7)	2.24 (95%CI: 1.87-2.68)	2.29 (95%CI: 1.95-2.70)
Palliative care	464	96	20.7% (95%CI: 17.1-24.7)	1.84 (95%CI: 1.38-2.44)	1.97 (95%CI: 1.50-2.56)
Sub-intensive care	191	21	11.0% (95%CI: 6.94-16.3)	1.68 (95%CI: 1.04-2.74)	0.93 (95%CI: 0.56-1.47)
Rehabilitation	3840	654	17.0% (95%CI: 15.9-18.3)	1.59 (95%CI: 1.33-1.91)	1.55 (95%CI: 1.31-1.83)
Hospital emergency department	2703	448	16.6% (95%CI: 15.2-18.0)	1.56 (95%CI: 1.29-1.89)	1.50 (95%CI: 1.26-1.79)
Cardiac surgery	579	81	14.0% (95%CI: 11.3-17.1)	1.56 (95%CI: 1.17-2.08)	1.23 (95%CI: 0.93-1.61)
Gastroenterology/Hepatology	221	31	14.0% (95%CI: 9.73-19.3)	1.46 (95%CI: 0.95-2.22)	1.23 (95%CI: 0.81-1.82)
Dermatology	126	18	14.3% (95%CI: 8.69-21.6)	1.46 (95%CI: 0.85-2.48)	1.26 (95%CI: 0.73-2.06)
Nephrology/Dialysis	1645	248	15.1% (95%CI: 13.4-16.9)	1.40 (95%CI: 1.13-1.73)	1.34 (95%CI: 1.10-1.63)
Oncology	1217	141	11.6% (95%CI: 9.84-13.5)	1.39 (95%CI: 1.09-1.77)	0.99 (95%CI: 0.79-1.24)
Cardiology	2031	298	14.7% (95%CI: 13.2-16.3)	1.38 (95%CI: 1.12-1.69)	1.30 (95%CI: 1.07-1.57)
Infectious diseases	590	70	11.9% (95%CI: 9.37-14.8)	1.36 (95%CI: 1.01-1.83)	1.01 (95%CI: 0.76-1.35)
Respiratory medicine	591	84	14.2% (95%CI: 11.5-17.3)	1.34 (95%CI: 1.01-1.78)	1.25 (95%CI: 0.95-1.63)
Endocrinology/Diabetes	137	16	11.7% (95%CI: 6.82-18.3)	1.31 (95%CI: 0.76-2.29)	1.00 (95%CI: 0.56-1.66)
Neurology	1173	158	13.5% (95%CI: 11.6-15.6)	1.26 (95%CI: 0.99-1.59)	1.17 (95%CI: 0.94-1.46)
Geriatric medicine	583	100	17.2% (95%CI: 14.2-20.5)	1.26 (95%CI: 0.96-1.66)	1.56 (95%CI: 1.20-2.02)
Neurosurgery	629	77	12.2% (95%CI: 9.78-15.1)	1.26 (95%CI: 0.94-1.69)	1.05 (95%CI: 0.79-1.38)
Urology	806	129	16.0% (95%CI: 13.5-18.7)	1.25 (95%CI: 0.97-1.62)	1.44 (95%CI: 1.13-1.82)
Outpatient facilities/Domiciliary care	606	110	18.2% (95%CI: 15.2-21.5)	1.17 (95%CI: 0.90-1.53)	1.67 (95%CI: 1.30-2.15)
Radiology/Radiotherapy	3584	390	10.9% (95%CI: 9.88-11.9)	1.11 (95%CI: 0.91-1.34)	0.92 (95%CI: 0.77-1.10)
Orthopedics/Traumatology	1819	242	13.3% (95%CI: 11.8-15.0)	1.11 (95%CI: 0.89-1.37)	1.16 (95%CI: 0.95-1.41)
Laboratory medicine	2690	279	10.4% (95%CI: 9.24-11.6)	1.06 (95%CI: 0.86-1.30)	0.87 (95%CI: 0.72-1.05)
Surgery	6650	813	12.2% (95%CI: 11.4-13.0)	1.05 (95%CI: 0.87-1.25)	1.05 (95%CI: 0.89-1.24)
Ophthalmology	721	74	10.3% (95%CI: 8.15-12.7)	0.96 (95%CI: 0.72-1.30)	0.86 (95%CI: 0.64-1.14)
Hygiene/Epidemiology	968	106	11.0% (95%CI: 9.05-13.1)	0.95 (95%CI: 0.73-1.23)	0.92 (95%CI: 0.72-1.18)
Paediatric psychiatry	872	87	9.98% (95%CI: 8.07-12.2)	0.94 (95%CI: 0.71-1.24)	0.83 (95%CI: 0.63-1.08)
Obstetrics/gynecology	2935	288	9.81% (95%CI: 8.76-10.9)	0.93 (95%CI: 0.75-1.13)	0.82 (95%CI: 0.68-0.99)
Hematology	718	54	7.52% (95%CI: 5.70-9.70)	0.92 (95%CI: 0.66-1.26)	0.61 (95%CI: 0.44-0.83)
Psychiatry	1415	146	10.3% (95%CI: 8.78-12.0)	0.88 (95%CI: 0.69-1.12)	0.87 (95%CI: 0.69-1.08)
Pediatrics	2735	240	8.78% (95%CI: 7.74-9.90)	0.88 (95%CI: 0.71-1.08)	0.73 (95%CI: 0.59-0.88)
Virology/Microbiology	319	25	7.84% (95%CI: 5.14-11.4)	0.87 (95%CI: 0.55-1.35)	0.64 (95%CI: 0.41-0.97)
Pre-hospital emergency medical services	135	16	11.9% (95%CI: 6.93-18.5)	0.87 (95%CI: 0.50-1.50)	1.01 (95%CI: 0.57-1.69)
General Practitioner	1083	138	12.7% (95%CI: 10.8-14.9)	0.83 (95%CI: 0.65-1.06)	1.1 (95%CI: 0.88-1.38)
Intensive care	3188	260	8.16% (95%CI: 7.23-9.16)	0.82 (95%CI: 0.67-1.01)	0.67 (95%CI: 0.55-0.81)
Histology/Anatomical pathology	705	59	8.37% (95%CI: 6.43-10.7)	0.71 (95%CI: 0.52-0.97)	0.69 (95%CI: 0.51-0.93)
Family pediatrician	140	14	10.0% (95%CI: 5.58-16.2)	0.59 (95%CI: 0.33-1.06)	0.84 (95%CI: 0.45-1.43)
Prison medicine	134	15	11.2% (95%CI: 6.40-17.8)	0.56 (95%CI: 0.31-0.98)	0.95 (95%CI: 0.52-1.61)
Medical Device Sterilization	397	25	6.30% (95%CI: 4.12-9.16)	0.54 (95%CI: 0.34-0.84)	0.51 (95%CI: 0.32-0.76)
Plastic surgery	168	8	4.76% (95%CI: 2.08-9.17)	0.49 (95%CI: 0.23-1.01)	0.38 (95%CI: 0.17-0.73)
Forensic medicine	155	7	4.52% (95%CI: 1.83-9.08)	0.40 (95%CI: 0.19-0.88)	0.36 (95%CI: 0.15-0.72)
Other clinical units§	4271	465	10.9% (95%CI: 9.97-11.9)	1.06 (95%CI: 0.88-1.27)	0.92 (95%CI: 0.78-1.10)
Other non-clinical units#	22244	2513	11.3% (95%CI: 10.9-11.7)	0.98 (95%CI: 0.84-1.15)	0.96 (95%CI: 0.83-1.12)

¶ estimates adjusted for model covariates

† crude estimates

§ aggregation of operating units dedicated to patient care with less than 200 employees

# administrative and support units entirely consisting of non-clinical staff

© 2021 Poletti P et al. *JAMA Network Open.*

eTable 3. Seroprevalence of Infection and Overall Hospitalization Incidence by Province, February to September 2020

Province	Tested for IgG antibodies	IgG positive	Seroprevalence	Hospitalized cases per 10,000 inhabitants
Bergamo	6291	1967	31.3% (95%CI: 30.1-32.4)	78.6
Cremona	4054	773	19.1% (95%CI: 17.9-20.3)	100.6
Lodi	2198	387	17.6% (95%CI: 16-19.3)	92.8
Brescia	11649	1992	17.1% (95%CI: 16.4-17.8)	74.7
Lecco	1446	164	11.3% (95%CI: 9.75-13.1)	42.8
Pavia	6905	750	10.9% (95%CI: 10.1-11.6)	48.1
Como	462	48	10.4% (95%CI: 7.76-13.5)	22.3
Mantova	4061	410	10.1% (95%CI: 9.19-11.1)	29.3
Sondrio	2140	208	9.72% (95%CI: 8.50-11.1)	34.7
Milano	31551	2676	8.48% (95%CI: 8.18-8.79)	32.9
Varese	4977	339	6.81% (95%CI: 6.13-7.55)	16.5
Monza-Brianza	5969	401	6.72% (95%CI: 6.10-7.38)	31.3

© 2021 Poletti P et al. *JAMA Network Open.*

eTable 4. Seroprevalence and Odds Ratios of Infection by Age Group and Sex

	**Tested for IgG antibodies**	**IgG positive**	**Seroprevalence**	**aOR of infection¶**	**cOR of infection†**
**Age***					
<50y	40500	5019	12.4% (95%CI: 12.1-12.7)	reference	reference
50y+	41203	5096	12.4% (95%CI: 12.1-12.7)	1.03 (95%CI: 0.99-1.08)	1.00 (95%CI: 0.96-1.04)
**Sex**					
Female	58912	7298	12.4% (95%CI: 12.1-12.7)	reference	reference
Male	22791	2817	12.4% (95%CI: 11.9-12.8)	1.08 (95%CI: 1.03-1.13)	1.00 (95%CI: 0.95-1.04)

* age groups were defined on the basis of the median age of HCWs in the considered sample

¶ estimates adjusted for model covariates

† crude estimates

© 2021 Poletti P et al. *JAMA Network Open.*

eTable 5. Hospital Admission by Age Group, February to September 2020

Age group	Number of COVID-19 patients admitted to hospital (%)
0-19	447 (1.0)
20-39	2,879 (6.2)
40-49	4,218 (9.1)
50-59	7,729 (16.6)
60-69	8,796 (18.9)
70-79	10,974 (23.6)
80+	11,510 (24.7)

© 2021 Poletti P et al. *JAMA Network Open.*

eTable 6. Seroprevalence and Odds Ratios by Professional Category Considering 1 Overall Surgical Unit and 1 Overall Internal Medicine Subspeciality Unit

Professional category	Tested for IgG antibodies	IgG positive	Seroprevalence	aOR of infection¶	cOR of infection†
Administrative	8135	940	11.6% (95%CI: 10.9-12.3)	reference	reference
Healthcare assistants	7549	1184	15.7% (95%CI: 14.9-16.5)	1.48 (95%CI: 1.33-1.65)	1.42 (95%CI: 1.30-1.56)
Nurses	29200	3905	13.4% (95%CI: 13.0-13.8)	1.28 (95%CI: 1.17-1.41)	1.18 (95%CI: 1.10-1.28)
Physicians	13915	1575	11.3% (95%CI: 10.8-11.9)	1.11 (95%CI: 1.00-1.23)	0.98 (95%CI: 0.89-1.06)
Medical students	619	55	8.89% (95%CI: 6.76-11.4)	0.96 (95%CI: 0.71-1.30)	0.74 (95%CI: 0.55-0.98)
Laboratory personnel	346	28	8.09% (95%CI: 5.44-11.5)	0.70 (95%CI: 0.46-1.05)	0.67 (95%CI: 0.44-0.97)
Radiographers	246	21	8.54% (95%CI: 5.36-12.8)	0.63 (95%CI: 0.39-1.02)	0.71 (95%CI: 0.44-1.10)
Other	21693	2407	11.1% (95%CI: 10.7-11.5)	0.98 (95%CI: 0.90-1.08)	0.95 (95%CI: 0.88-1.04)

¶ estimates adjusted for model covariates

† crude estimates

© 2021 Poletti P et al. *JAMA Network Open.*

eTable 7. Seroprevalence and Odds Ratios by Age Group and Sex Considering 1 Overall Surgical Unit and 1 Overall Internal Medicine Subspeciality Unit

	Tested for IgG antibodies	IgG positive	Seroprevalence	aOR of infection¶	cOR of infection†
**Age***					
<50y	40500	5019	12.4% (95%CI: 12.1-12.7)	reference	reference
50y+	41203	5096	12.4% (95%CI: 12.1-12.7)	1.03 (95%CI: 0.99-1.08)	0.99 (95%CI: 0.95-1.04)
**Sex**					
Female	58912	7298	12.4% (95%CI: 12.1-12.7)	Reference	reference
Male	22791	2817	12.4% (95%CI: 11.9-12.8)	1.08 (95%CI: 1.03-1.14)	0.99 (95%CI: 0.95-1.04)

* age groups were defined on the basis of the median age of HCWs in the considered sample

¶ estimates adjusted for model covariates

† crude estimates

© 2021 Poletti P et al. *JAMA Network Open.*

eTable 8. Seroprevalence and Odds Ratios by Operational Unit Considering 1 Overall Surgical Unit and 1 Overall Internal Medicine Subspeciality Unit

Operational unit	Tested for IgG antibodies	IgG positive	Seroprevalence	aOR of infection¶	cOR of infection†
Telephone operators	1819	213	11.7% (95%CI: 10.3-13.3)	reference	reference
Internal medicine	3598	838	23.3% (95%CI: 21.9-24.7)	2.24 (95%CI: 1.87-2.68)	2.29 (95%CI: 1.95-2.70)
Palliative care	464	96	20.7% (95%CI: 17.1-24.7)	1.84 (95%CI: 1.38-2.44)	1.97 (95%CI: 1.50-2.56)
Sub-intensive care	191	21	11.0% (95%CI: 6.94-16.3)	1.68 (95%CI: 1.03-2.73)	0.93 (95%CI: 0.56-1.47)
Rehabilitation	3840	654	17.0% (95%CI: 15.9-18.3)	1.59 (95%CI: 1.33-1.91)	1.55 (95%CI: 1.31-1.83)
Hospital emergency department	2703	448	16.6% (95%CI: 15.2-18)	1.56 (95%CI: 1.29-1.89)	1.50 (95%CI: 1.26-1.79)
Infectious diseases	590	70	11.9% (95%CI: 9.37-14.8)	1.36 (95%CI: 1.01-1.83)	1.01 (95%CI: 0.75-1.35)
Respiratory medicine	591	84	14.2% (95%CI: 11.5-17.3)	1.34 (95%CI: 1.01-1.78)	1.25 (95%CI: 0.94-1.63)
Internal medicine sub-specialties*	6974	940	13.5% (95%CI: 12.7-14.3)	1.33 (95%CI: 1.12-1.59)	1.17 (95%CI: 1.00-1.38)
Neurology	1173	158	13.5% (95%CI: 11.6-15.6)	1.26 (95%CI: 1.00-1.59)	1.17 (95%CI: 0.94-1.46)
Outpatient facilities/Domiciliary care	606	110	18.2% (95%CI: 15.2-21.5)	1.17 (95%CI: 0.90-1.52)	1.67 (95%CI: 1.30-2.15)
Radiology/Radiotherapy	3584	390	10.9% (95%CI: 9.88-11.9)	1.11 (95%CI: 0.91-1.34)	0.92 (95%CI: 0.77-1.1)
Surgery	10651	1350	12.7% (95%CI: 12.0-13.3)	1.10 (95%CI: 0.92-1.30)	1.09 (95%CI: 0.94-1.28)
Laboratory medicine	2690	279	10.4% (95%CI: 9.24-11.6)	1.06 (95%CI: 0.86-1.30)	0.87 (95%CI: 0.72-1.05)
Hygiene/Epidemiology	968	106	11.0% (95%CI: 9.05-13.1)	0.95 (95%CI: 0.73-1.23)	0.92 (95%CI: 0.72-1.18)
Obstetrics/gynecology	2935	288	9.81% (95%CI: 8.76-10.9)	0.92 (95%CI: 0.75-1.13)	0.82 (95%CI: 0.68-0.99)
Psychiatry	2287	233	10.2% (95%CI: 8.98-11.5)	0.90 (95%CI: 0.73-1.11)	0.85 (95%CI: 0.70-1.04)
Pediatrics	2735	240	8.78% (95%CI: 7.74-9.90)	0.87 (95%CI: 0.71-1.08)	0.72 (95%CI: 0.59-0.88)
Virology/Microbiology	319	25	7.84% (95%CI: 5.14-11.4)	0.87 (95%CI: 0.56-1.35)	0.64 (95%CI: 0.40-0.97)
Pre-hospital emergency medical services	135	16	11.9% (95%CI: 6.93-18.5)	0.86 (95%CI: 0.49-1.5)	1.01 (95%CI: 0.56-1.69)
General Practitioner	1083	138	12.7% (95%CI: 10.8-14.9)	0.82 (95%CI: 0.64-1.06)	1.10 (95%CI: 0.87-1.38)
Intensive care	3188	260	8.16% (95%CI: 7.23-9.16)	0.81 (95%CI: 0.66-1.01)	0.67 (95%CI: 0.55-0.811)
Histology/Anatomical pathology	705	59	8.37% (95%CI: 6.43-10.7)	0.71 (95%CI: 0.51-0.97)	0.68 (95%CI: 0.50-0.92)
Family pediatrician	140	14	10.0% (95%CI: 5.58-16.2)	0.58 (95%CI: 0.32-1.05)	0.83 (95%CI: 0.45-1.43)
Prison medicine	134	15	11.2% (95%CI: 6.40-17.8)	0.55 (95%CI: 0.31-0.98)	0.95 (95%CI: 0.52-1.61)
Medical Device Sterilization	397	25	6.30% (95%CI: 4.12-9.16)	0.54 (95%CI: 0.34-0.84)	0.50 (95%CI: 0.32-0.76)
Forensic medicine	155	7	4.52% (95%CI: 1.83-9.08)	0.40 (95%CI: 0.18-0.87)	0.35 (95%CI: 0.15-0.71)
Other clinical units §	4804	525	10.9% (95%CI: 10.1-11.8)	1.05 (95%CI: 0.87-1.27)	0.92 (95%CI: 0.78-1.1)
Other non-clinical units#	22244	2513	11.3% (95%CI: 10.9-11.7)	0.98 (95%CI: 0.83-1.15)	0.96 (95%CI: 0.82-1.12)

¶ estimates adjusted for model covariates

† crude estimates

§ aggregation of operating units dedicated to patient care with less than 200 employees

# administrative and support units entirely consisting of non-clinical staff

* aggregation of hospital wards dedicated to internal medicine sub-specialties

© 2021 Poletti P et al. *JAMA Network Open.*
